# Patient to patient transmission of hepatitis B virus: a systematic review of reports on outbreaks between 1992 and 2007

**DOI:** 10.1186/1741-7015-7-15

**Published:** 2009-04-08

**Authors:** Simone Lanini, Vincenzo Puro, Francesco N Lauria, Francesco M Fusco, Carla Nisii, Giuseppe Ippolito

**Affiliations:** 1 Istituto Nazionale per le Malattie Infettive, Lazzaro Spallanzani-Roma, Via Portuense, 00149 Rome, Italy

## Abstract

**Background:**

Hepatitis B outbreaks in healthcare settings are still a serious public health concern in high-income countries. To elucidate the most frequent infection pathways and clinical settings involved, we performed a systematic review of hepatitis B virus outbreaks published between 1992 and 2007 within the EU and USA.

**Methods:**

The research was performed using two different databases: the PubMed Database and the Outbreak Database, the worldwide database for nosocomial outbreaks. Selection of papers was carried out using the Quorom algorithm, and to avoid selection biases, the inclusion criteria were established before the articles were identified.

**Results:**

Overall, 30 papers were analyzed, reporting on 33 hepatitis B virus outbreaks that involved 471 patients, with 16 fatal cases. Dialysis units accounted for 30.3% of outbreaks followed by medical wards (21.2%), nursing homes (21.2%), surgery wards (15.2), and outpatient clinics (12.1%). The transmission pathways were: multi-vial drugs (30.3%), non-disposable multi-patient capillary blood sampling devices (27.2%), transvenous endomyocardial biopsy procedures (9.1%), and multiple deficiencies in applying standard precautions (9.1%).

**Conclusion:**

The analysis of transmission pathways showed that some breaches in infection control measures, such as administration of drugs using multi-vial compounds and capillary blood sampling, are the most frequent routes for patient-to-patient transmission of hepatitis B virus. Moreover some outbreak reports underlined that heart-transplant recipients are at risk of contracting hepatitis B virus infection during the transvenous endomyocardial biopsy procedure through indirect contact with infected blood as a result of environmental contamination. To prevent transmission, healthcare workers must adhere to standard precautions and follow fundamental infection control principles, such as the use of sterile, single-use, disposable needles and avoiding the use of multi-vial compounds in all healthcare settings including outpatient settings.

## Background

Despite a reduction of newly acquired hepatitis B virus (HBV) infections since the introduction of vaccination in early 1990s, HBV remains an important cause of liver disease in developed countries. Moreover, the virus has long been recognized as one of the most insidious viral agents within healthcare settings (HCS), and in fact a number of HBV outbreaks in HCS are reported yearly in the USA and EU.

In HCS, HBV is mainly a blood-borne infection transmitted to susceptible patients either from an infected healthcare worker (HCW) (professional-to-patient transmission) or from another infected patient (patient-to-patient transmission) [1].

The professional-to-patient transmission has been widely investigated and is currently well accepted to be generally related to exposure-prone procedures performed by a viremic HCW; comprehensive guidelines are available on the matter [2,3]. On the other hand, no comprehensive systematic reviews on HBV patient-to-patient transmission have been published, even though patient-to-patient transmission of HBV is still a serious problem often involving subjects suffering from other severe conditions.

To describe the most frequent pathways of patient-to-patient transmission and clinical conditions associated with the infection, and to make a comparison between European and American outbreaks, we performed a systematic review of reports published in the EU and USA between 1992 and 2007.

## Methods

### Search strategy

We searched the PubMed Database [4] and the Outbreak Database, the worldwide database for nosocomial outbreaks supported by the Charité – University Medicine Berlin [5]. The search on the PubMed electronic database was limited to studies published from 1 January 1992 to 31 December 2007, human subjects and English language. The mesh-terms 'hepatitis B/transmission', 'hepatitis B virus', 'cross infection', 'disease outbreaks' and 'iatrogenic disease' were used to obtain the search string. The search on the Outbreak Database was made using the key word 'hepatitis B' according to the provider's instructions; reports published before 1 January 1992 or after 31 December 2007 and the ones already retrieved through PubMed were manually excluded.

### Selection criteria

Inclusion criteria were established before articles were identified to avoid selection biases. Only outbreak investigation reports describing patient-to-patient transmission of HBV in HCS in the USA and EU between 1992 and 2007 were included. We considered 'outbreak investigation reports' as only those papers reporting a description of the population and setting, and the outline of the epidemiological investigation performed. We included only cases of HBV infection that were HBsAg-positive, as minimum criteria. HCW-to-patient transmission had to have been ruled out either because the presence of an hepatitis B surface antigen (HBsAg)-positive HCW was excluded, or by identifying the patient index case through molecular characterization of the epidemic cluster(s).

We decided to consider only the USA and EU because they represent two homogeneous areas with high health standards and comparable sociodemographic indicators but different healthcare systems (i.e.: public or a public-private mix for the EU, mainly private for the USA). Furthermore, only papers published between 1992 and 2007 (including outbreaks that occurred between 1990 and 2004) were considered because this period is assumed to be rather homogenous and highly significant with regard to HBV epidemiology, standards of care, availability of diagnostic technologies, and medical devices. We also recorded the time between the end of the outbreak and the publication of the selected papers, as a further control for a possible bias due to publication delays.

**Figure 1:**
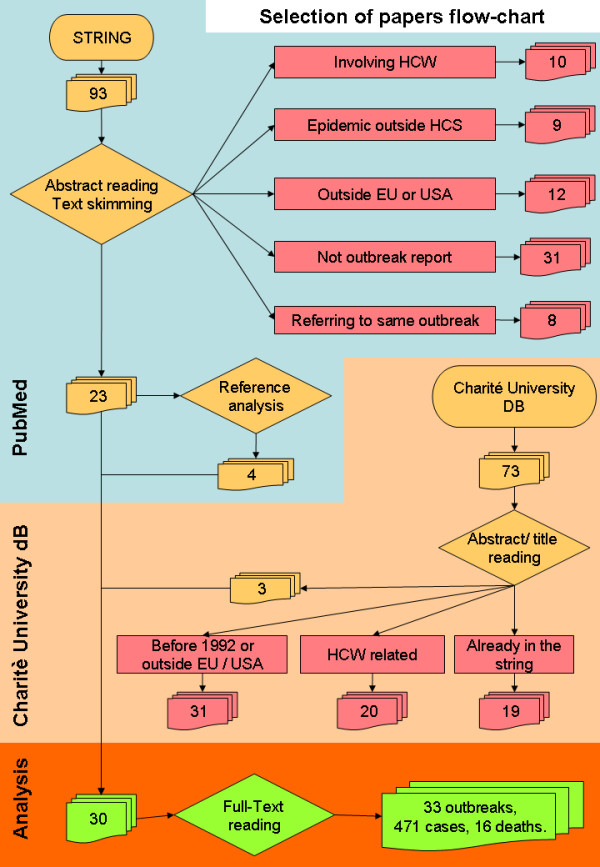
**The Quorom-based algorithm used to carry out the selection of papers**.

The selection of papers was carried out with a Quorom-based algorithm [6,7] (Figure 1) by two authors (SL and VP), all titles, abstracts (if available) and full-text versions were examined. To guarantee transparency throughout the selection process, all excluded papers were ranked according to five main exclusion criteria:

1. investigation performed outside the EU or USA (reports from countries that joined the EU after 1990 were included only if the outbreak happened after that year);

2. outbreaks in which HCWs were identified as the source of infection;

3. outbreaks outside a HCS;

4. papers that were a partial report of outbreaks already selected;

5. papers which did not fit our definition of 'outbreak report' (e.g. reviews, editorials, etc).

Excluded papers were assigned only one label, however, some papers were excluded for more than one criterion. Finally, the lists of references of selected papers were screened for additional data.

As required by the editorial policy the "Quorum checklist" for systematic reviews is reported in additional file 1.

### Data extraction

Data were extracted by two authors (SL and VP). They were not blinded to the names of the authors, institutions, journal of publication or study results. Information was collected on the following: country where the outbreak happened; publication date; date of onset of the outbreak (i.e.: for in-patient settings the day of admission of the index case, or the day of first referral for care of the index case for outpatient settings); date of the end of the outbreak (i.e.: the onset of the last case); type of healthcare facility; population size; population characteristics (i.e.: the main clinical features of patients admitted to the unit); total number of cases; number of deaths due to the infection; number of non-susceptible patients (i.e.: patients with markers of previous infection or vaccination, which was considered to be zero if authors did not report any information); possible/probable risk factors and transmission pathway.

With regard to laboratory methods, we considered as molecular epidemiology techniques only the characterization of infection cluster(s) through polymerase chain reaction amplification of one or more selected region(s) of the viral genome. Genotyping and other methods of cluster characterization, including serological characterizations, such as sub-typing, were considered standard epidemiology techniques.

### Data analysis

Five parameters were considered in the study: total number of cases; duration of the outbreak (i.e.: time in months between the day of onset and day of end of the outbreak or as reported by the authors); attack rate (AR) (i.e.: number of cases/susceptible subjects); fatality rate (FR) (i.e.: deaths/total of cases); time to publication (i.e.: time in months between the day of end of outbreak and publication).

Variations of median values between different outbreaks were analyzed in light of four different outbreak characteristics:

1. location, i.e.: EU versus USA;

2. HCS, i.e.: dialysis units versus others;

3. transmission pathways, i.e.: use of multi-vial drugs, capillary blood sampling (CBS), multiple deficiencies in standard precautions (i.e.: circumstances in which the authors found a number of inaccuracies in the application of standard precautions but did not correlate the outbreak with one in particular); transvenous endomyocardial biopsy (TEB), blood products, and undefined (i.e.: cases in which the authors failed to point out any evident inaccuracy in the application of standard precautions);

4. patients' clinical condition, i.e.: neoplasm versus others.

Moreover, we analyzed the use of molecular epidemiology techniques throughout the period.

The analysis was carried out by comparing median values using the outbreaks as the statistical units. The variance of the medians of each of the five parameters was separately tested for the four outbreak characteristics considered; *P *values (significant if <0.05) were calculated with the Mann-Whitney U test for outbreak characteristics with two ranks, and with the Kruskal-Wallis one-way analysis of variance for outbreak characteristics with more than two ranks. The analysis was carried out by Epi-info 3.5.1.

## Results

The PubMed search resulted in 93 papers, 23 of which were judged to meet the inclusion criteria and were considered in this review; four additional papers were retrieved from the references. In addition, consultation of the Outbreak Database provided three more papers which met the inclusion criteria. In this way, 30 papers were analyzed in total, amounting to 33 HBV outbreaks, involving 471 patients with 16 fatal cases (Figure 1).

The list of all outbreaks including main features and references is reported in Additional file 2.

Dialysis units were involved in 10 out of 33 reported outbreaks (30.3%), medical wards and nursing home services in seven outbreaks (21.2%) each, surgery wards in five (15.2%) and outpatient clinics in four (12.1%) (Table 1). Hematology units were the most recurrent among medical wards, and heart-transplant units were the most frequent among surgery wards with three outbreaks each (hematology, see rows 4, 18, 28 in Additional file 2; heart-transplant units, see rows 2, 13, 31 in Additional file 2).

**Table 1:** Summary of the most frequent transmission pathways and most frequent healthcare settings involved.

	**Transmission pathways**						
**Healthcare setting**	**Multi-vials**	**Capillary blood sampling**	**Multiple deficiencies in standard precautions**	**Transvenous biopsy**	**Blood products**	**Undefined**	**Total**

Dialysis	5	-	1	-	1	3	10

Medicine	3	3	-	-	1	-	7

Nursing home	-	6	-	-	-	1	7

Surgery	-	-	-	3	-	2	5

Outpatient clinics	2	-	2	-	-	-	4

Total	10	9	3	3	2	6	33

The majority of the outbreaks originated among patients already affected by one or more underlying conditions causing some degree of immunodepression, such as end-stage renal diseases (ten investigations, 30.3%), diabetes (nine investigations, 27.3%), neoplasms (four investigations, 12.1%), heart transplantation (three investigations, 9.1%), and only seven outbreaks (21.2%) affected patients with no reported immunodepression.

A specific transmission pathway was found in 27 out of 33 outbreaks (Table 1), no clear explanation was given in the remaining six (18.2%). The most frequent transmission pathways were: multi-vial drugs in ten reports (30.3%), non-disposable multi-patient CBS devices in nine reports (27.3%) and TEB in three reports (9.1%). Multiple deficiencies in applying standard precautions were identified in three reports (9.1%). Transmission of HBV through contaminated blood products was reported twice (6.1%): the first event was related to the infusion of autologous hematopoietic stem cells extrinsically contaminated (see row 4 in Additional file 2) and the other was linked with heterologous fresh-frozen plasma from a donor with occult HBV infection. (see row 12 in Additional file 2).

Overall, molecular epidemiologic techniques were used in 15 out of 33 investigations (45.5%), although the proportion of investigations using such technology steadily increased over the studied period, from only 22.2% between 1992 and 1996, 40.0% between 1997 and 2002 to 64.3% in the studies published between 2003 and 2007. Molecular technologies were used to confirm all outbreaks through the determination of phylogenetic identity between the virus strains infecting patients, but, apart from one case (see row 4 in Additional file 2), no environmental samples were investigated in this way.

Sixteen out of 33 outbreaks were from EU countries: four from the UK, three from Italy, two from Germany, two from Sweden and one each from Belgium, Spain, France, Denmark and the Netherlands; the remaining 17 outbreaks were from the USA. No significant differences in the main epidemiologic parameters, such as number of cases, AR, FR, and outbreak duration, were found between EU and USA outbreaks; of note, the median time to publication was 39.5 months for EU and 22 months for USA reports (*P *= 0.0068).

ARs were calculated in 29 outbreaks (no data were provided on the exposed population in four reports). Although the ARs ranged widely between 0.66% and 70.00% (median 8.9%), the variance of median values, according to the outbreak characteristics, was not statistically significant.

Sixteen deaths were reported in five out of the 33 outbreaks. Two out of those five, accounting for 11 out of 16 deaths, occurred among patients with neoplasms, who reported a significantly higher median FR (*P *= 0.0215).

The outbreak duration was estimated for each event and, according to the wide range of clinical and epidemiologic conditions reported, it broadly ranged between 1 and 145 months (median 6 months). When the different transmission pathways were considered, it was found that the longest outbreak duration affected patients having undergone TEB (median 124 months; *P *= 0.0303); whereas when studying the different HCS, it emerged that outbreaks which occurred in dialysis units had the shortest duration (median 3.5 months; *P *= 0.0248) (Table 2).

**Table 2:** Details of outbreaks.

**Outbreak characteristics**		**Number of outbreaks**	**Duration of outbreaks (months)**	**p**	**Cases** **(number)**	**p**	**Fatality rate** **(%)**	p
								
			**Median value** **(range)**		**Median value (range)**		**Median value (range)**	
Location	EU	16	11 (1–145)	0.3849^a^	5 (1–86)	0.4815^a^	0 (0–100)	0.4705^a^
						
	USA	17	5 (2–40)		9 (1–38)		0 (0–16.7)	

Clinical condition	Neoplasia	4	10 (3–25)	0.5059^a^	6 (2–11)	0.4391^a^	40.9 (0–100)	**0.0215^a^**
						
	Other	29	5(1–145)		8 (1–86)		0 (0–40.0)	

Transmission pathways	Multi-vials	10	3.5 (1–14)		6.5 (2–29)		0 (0–81.8)	
						
	Capillary blood sampling.	9	9 (2–16)		11 (3–27)		0 (0–40.0)	
						
	Standard^†^	3	24 (4–40)		31 (14–38)		0(-)	
						
	Transvenous biopsy	3	124 (54–145)	**0.0303^b^**	63 (20–86)	**0.0012^b^**	0(-)	0.6384^b^
						
	Blood product	2	13.5 (2–25)		3 (1–5)		0(-)	
						
	Undefined	6	4.5 (2–17)		2.5 (1–6)		0 (0–100)	

Setting	Dialysis	10	3.5 (2–17)		4 (1–14)		0 (-)	
						
	Others	23	9 (1–145)	**0.0248^a^**	11 (1–86)	**0.0240^a^**	0 (0–100)	0.1165^a^

	Total	33	6 (1–145)	-	7 (1–86)	-	0(0–100)	-

^a^Mann-Whitney U test.

^b^Kruskal-Wallis one-way analysis of variance by ranks.

† Multiple deficiencies in standard precautions; standard precautions as defined by the Centers for Disease Control and Prevention [10].

The total number of cases was given for all reports and ranged between 1 and 86 (median 7). The highest number of cases was found in the outbreaks associated with TEB and with multiple deficiencies in standard precautions (median 63 and 31, respectively; *P *= 0.0012); considering the different HCS, the outbreaks that occurred in dialysis units had the lowest number of cases (median four; *P *= 0.0240) (Table 2).

## Discussion

HBV outbreaks occurring within HCS are still a serious public health issue in high-income countries involving mainly patients already affected by severe conditions, such as chronic renal failure, diabetes, and cancer, or patients who have undergone solid organ transplant.

This review analyzed only published events and does not draw a reliable picture of the actual burden of HBV outbreaks within HCS. The true incidence of events, the overall numbers of cases and deaths are very difficult to identify given the limitations due to publication biases, possible under-reporting of events, and the sensitivity limits of the systematic research. Moreover, since there were no specific guidelines for reporting outbreak investigations within HCS until 2007 [8], the reports had very different formats in which relevant data (e.g. the true number of exposed patients or the total number of non-susceptible patients) were omitted in some cases.

Notwithstanding these limitations, this review highlights several topical factors. Firstly, we found that dialysis units accounted for the highest number of outbreaks (10 out of 33), and that such outbreaks were the ones with the shortest duration and the fewest number of cases. These data might be explained by the fact that, in both the USA and most EU countries, dialysis units have widely improved and mandatory protocols for serological surveillance of blood-borne infections, such as HBV and hepatitis C virus (HCV), which enabled healthcare providers to promptly identify also asymptomatic cases; the consciousness of the risk of HBV transmission might also explain the higher frequency of reporting in dialysis units than in other settings. We also found that the highest number of such outbreaks was associated with the use of multi-vials, which is not unexpected. HBV outbreaks within dialysis facilities have long been recognized as a serious problem [9,10], and the evidence that dialyzed patients have a higher prevalence of blood-borne infections (e.g. HBV and HCV) [11] has sometimes been reported as an indirect proof of HBV transmission during the dialysis process. However the contamination of blood during dialysis is hardly imaginable when disposable dialyzer circuits and machines with electronic fail-safe systems are used. Consistent with the Centers for Disease Control and Prevention (CDC) data, the results of this review strengthen the idea that dialysis itself is nowadays a rather safe procedure and that outbreaks are largely due to 'substantial deficiencies in recommended infection control practice, such as the use of multi-vial drugs, as well as failure to vaccinate hemodialysis patients against hepatitis B' [12]. It is noteworthy that the latest revision of the 'Guideline for Isolation Precautions: Preventing Transmission of Infectious Agents in Healthcare Settings' released by CDC in June 2007 [1] has included specific recommendations against the use of multi-vial compounds to deal expressly with outbreaks of viral hepatitis.

Patients with diabetes were found to be involved in HBV outbreaks as a consequence of the habit of performing CBS using non-disposable multi-patient devices. CBS is a rapid and cost-effective tool for glycemia control in diabetic individuals both at home and during hospital stays. Non-disposable multi-patient CBS devices have frequently been believed to be safe by virtue of the sterility of their piercing compound (i.e. disposable lancets). Nevertheless transmission through the non-disposable components cannot be ruled out and such devices should be reserved only for personal use at home, and replaced by safety lancets in HCS. In fact, due to its resistance to environmental compounds [13], HBV can be transmitted through this route better than other blood-borne pathogens, such as human immunodeficiency virus (HIV) and HCV, and the risk of spreading HBV through CBS has been proposed since the early 1990s [14].

Heart-transplant recipients appeared also to be at increased risk of HBV infection, related to TEB. TEB has been accepted as an accurate method to evaluate the status of cardiac transplant rejection, and heart-transplant recipients usually undergo this procedure several times after transplantation [15]. In the reports analyzed here, infections are believed to have happened because of the common, procedure-generated spread of blood droplets, especially during purging of syringes and withdrawal of the catheter. Those droplets might contaminate unwrapped TEB material and the virus could be transmitted to the next patient. It is noteworthy that the spreading of blood-borne pathogens through the contamination of the healthcare environment has been supported by previous studies which demonstrated the presence of HBV in the apparently clean environment surrounding an HBsAg-positive patient after a vascular procedure [16-20] or dialysis [21], therefore, environmental contamination could play a role in outbreaks with unclear transmission pathways, in HCS such as dialysis units.

Due to the complexity of different biological patterns of HBV infection (e.g. occult hepatitis, HBsAg mutants and anti-HBs to HBsAg reversion in immunodepressed subjects), the risk of HBV transmission through unrecognized contaminated blood components is higher than the risk for other blood-borne pathogens such as HIV and HCV [22,23]. Here, we found two events of HBV transmission both involving an HBsAg-negative subject as index case.

In one outbreak five patients were infected after being transfused with autologous hematopoietic stem cells contaminated during the preservation procedure; it is to be underlined that HBV serological markers (i.e. HBsAg and anti-HBc) were negative in all patients at the time of stem cell harvesting, however the liquid nitrogen used for cryopreservation was found to be contaminated by HBV DNA and human DNA as a result of the rupture of one of the cryopreservation bags (see row 4 in Additional file 2). In the second event the infection was due to transfusion of fresh frozen plasma from a donor with occult hepatitis (see row 12 in Additional file 2).

Patients with neoplasms were found to have a higher FR, which is consistent with the evidence that such patients are more prone than others to die after the infection. In fact chemotherapy is known to enhance HBV replication and a number of cases of fulminant hepatitis have been described as the results of the reactivation of chronic infections or the reversing from anti-HBsAg to HBsAg-positive status.

Although a wide range of AR was observed, we did not identify any significant difference in AR median values between outbreaks according to the different characteristics of outbreaks considered. The lack of data on the infectivity of the index cases did not allow any inference about this issue.

No outbreaks associated with endoscopic procedures were found, and this is consistent with literature data. The absence of endoscopic transmission of HBV has been described in 12 reports studying 394 patients exposed to endoscopies that had recently been used on HBsAg-positive patients; none developed clinical hepatitis and 357 (91%) had serologic confirmation that they had not contracted HBV infection [24].

Moreover, two recent systematic reviews describing 140 [25] and 70 [26] endoscopic-related healthcare-associated infections between 1974 and 2004, respectively, underline that exogenous endoscopy-related infections are rare events and generally restricted to bacterial agents. Nevertheless these reviews reported three cases of HBV transmission [27-29] that were not included in our review (two which occurred before 1992 and one of uncertain relevance). However, even if there is no definitive evidence of acute HBV infection as a consequence of endoscopy, contamination with HBV DNA in gastrointestinal endoscope channels has been reported [30-32], therefore, reprocessing procedures of all endoscopic devices should always be carefully applied.

Overall no differences have been found between the EU and US reports, which indicate that no discrepancies exist with regard to the characteristics and management of the outbreaks in the USA and EU. It is noteworthy that since the beginning of the analysis, a rather evident disparity has been found between USA and EU papers, regarding the time between the end of the outbreak and publication. This significant difference might be due to the easier and faster publishing system in the USA, where outbreak reports are preliminarily published in the CDC weekly bulletin (i.e. *MMWR Morbidity and Mortality Weekly Report*), indexed since 1981, and eventually in peer-reviewed journals. Indeed, 12 out of 17 US outbreaks were published in *MMWR*.

In contrast, all but one (UK; see row 17 in Additional file 2) of EU reports have been published only in peer-reviewed journals. However, since March 2007, the European Centre for Disease Prevention and Control has also been publishing its official weekly bulletin (*Eurosurveillance*) and whether the availability of this new tool could impact on filling this gap will be evaluated in the near future.

## Conclusion

We have found that several breaches in infection control measures, related to some routine clinical practices thought to be risk-free (e.g. point of care blood glucose monitoring or preparation and administration of common parenteral drugs with multi-vial compounds) could result in patient-to-patient transmission of HBV within HCS. To prevent transmission of blood-borne pathogens, HCWs must adhere to standard precautions and follow fundamental infection-control principles, such as the use of sterile, single-use, disposable needles, and avoid the use of multi-vial compounds in all HCS including outpatient settings. These principles and practices need to be made explicit in institutional policies and reinforced through in-service education for all personnel involved in direct patient care.

## Abbreviations

AR: attack rate; CBS: capillary blood sampling; CDC: Centers for Disease Control and Prevention; FR: fatality rate; HBsAg: hepatitis B surface antigen; HBV: hepatitis B virus; HCS: healthcare settings; HCV: hepatitis C virus; HCW: healthcare workers; HIV: human immunodeficiency virus; *MMWR*: *MMWR Morb Mortal Wkly Rep*; TEB: transvenous endomyocardial biopsy.

## Competing interests

The authors declare that they have no competing interests.

## Authors' contributions

SL made substantial contributions to the conception and design of the study, acquisition, analysis and interpretation of data, was involved in drafting the manuscript and gave approval of the final version. VP made substantial contributions to the acquisition, analysis and interpretation of data, was involved in critically revising the manuscript for important intellectual content and gave approval of the final version. FNL, FMF, CN, and GI made substantial contributions to the analysis and interpretation of data, critically revised the manuscript for important intellectual content and gave approval of the final version.

## Pre-publication history

The pre-publication history for this paper can be accessed here:



## Supplementary Material

Additional file 1 **Quorom statement checklist**. This dataset includes the Quorum checklist as required by the editorial policies. Click here for file

Additional file 2 **Synopsis of analyzed outbreaks**. This dataset includes bibliographic references and the most relevant epidemiologic parameters. Click here for file
