# Supplementary material for: Patient to patient transmission of hepatitis B virus: a systematic review of reports on outbreaks between 1992 and 2007
Source: BMC Med. 2009 Apr 8;7:15. doi: 10.1186/1741-7015-7-15 (PMC2676313; doi:10.1186/1741-7015-7-15)
Supplement: Additional file 1 — Quorom statement checklist. This dataset includes the Quorum checklist as required by the editorial policies. [file 1741-7015-7-15-S1.rtf]

QUOROM statement checklist

_______________________________________________________________________________________________________________________________________________________________________________________

Heading 		Subheading 		Descriptor 								Reported? (Yes/No) 		Page number
-------------------	-----------------------------	-------------------------------------------------------------------------------------------------------------	---------------------------	-----------------------
Title 					Identify the report as a meta-analysis [or systematic review] of RCTs26			                Yes		              1
-------------------	----------------------------	-------------------------------------------------------------------------------------------------------------	---------------------------	-----------------------
Abstract					Use a structured format27								Yes		              2

Describe
Objectives 		The clinical question explicitly
Data sources 		The databases (ie, list) and other information sources
Review methods		The selection criteria (ie, population, intervention, outcome, and study design);
methods for validity assessment, data abstraction, and study characteristics, and
quantitative data synthesis in sufficient detail to permit replication
Results 			Characteristics of the RCTs included and excluded; qualitative and quantitative
findings (ie, point estimates and confidence intervals); and subgroup analyses
Conclusion 		The main results
------------------	----------------------------	---------------------------------------------------------------------------------------------------------------	----------------------------	-------------------------
Describe
------------------	----------------------------	----------------------------------------------------------------------------------------------------------------	-----------------------------	--------------------------
Introduction 				The explicit clinical problem, biological rationale for the intervention, and rationale for review	Yes			3
------------------	-----------------------------	----------------------------------------------------------------------------------------------------------------	------------------------------	--------------------------
Methods 		Searching 		The information sources, in detail28 (eg, databases, registers, personal files, expert		Yes		              4,5,6
informants, agencies, hand-searching), and any restrictions (years considered, publication
status,29 language of publication30,31)
Selection 			The inclusion and exclusion criteria (defining population, intervention, principal			Yes			
outcomes, and study design32
Validity assessment 	The criteria and process used (eg, masked conditions, quality assessment, and their findings33–36)	No			
Data abstraction 		The process or processes used (eg, completed independently, in duplicate)35,36		                Yes		
Study characteristics 	The type of study design, participants' characteristics, details of intervention, outcome		No			
definitions, &c,37 and how clinical heterogeneity was assessed
Quantitative data synthesis 	The principal measures of effect (eg, relative risk), method of combining results			Yes			
(statistical testing and confidence intervals), handling of missing data; how statistical
heterogeneity was assessed;38 a rationale for any a-priori sensitivity and subgroup analyses;
and any assessment of publication bias39
------------------	-----------------------------	---------------------------------------------------------------------------------------------------------------	------------------------------	--------------------------
Results 		Trial flow 			Provide a meta-analysis profile summarising trial flow (see figure)				Yes			7,8
Study characteristics	Present descriptive data for each trial (eg, age, sample size, intervention, dose, duration,
follow-up period)									Yes
Quantitative data synthesis 	Report agreement on the selection and validity assessment; present simple summary		Yes
results (for each treatment group in each trial, for each primary outcome); present data
needed to calculate effect sizes and confidence intervals in intention-to-treat analyses
(eg 2X2 tables of counts, means and SDs, proportions)

---------------------	----------------------------	-------------------------------------------------------------------------------------------------------------------	------------------------------	-------------------------
Discussion & 
conclusion				Summarise key findings; discuss clinical inferences based on internal and external validity;	Yes		         9,10,11,12
interpret the results in light of the totality of available evidence; describe potential
biases in the review process (eg, publication bias); and suggest a future research agenda

________________________________________________________________________________________________________________________________________________________
