# Supplementary material for: Patient to patient transmission of hepatitis B virus: a systematic review of reports on outbreaks between 1992 and 2007
Source: BMC Med. 2009 Apr 8;7:15. doi: 10.1186/1741-7015-7-15 (PMC2676313; doi:10.1186/1741-7015-7-15)
Supplement: Additional file 2 — Synopsis of analyzed outbreaks. This dataset includes bibliographic references and the most relevant epidemiologic parameters. [file 1741-7015-7-15-S2.rtf]

Annex 1 


Synopsis of analyzed outbreaks	
Outbreak	Autore	Journal	Pub. date	Country	Time 
to pub	Duration	HCS	Clinical conditions	Transmission	N.cases	N. deaths	AR%	FR%	Laboratory	
1	Polish LB	N Engl J Med	12/03/1992	USA	23	12	Medicine	Diabetes	CBS	27	0	7,92	0	SE	
2	Drescher J	J Hosp Infect.	01/02/1994	Germany	41	54	Surgery	Transplant	TEB	63	0	25.93	0	SE	
3	Roll M	J Hosp Infect	01/05/1995	Sweden	38	13	Dialysis	CRF	Undefined	4	0	8.89	0	ME	
4	Tedder RS	Lancet	15/06/1995	UK	14	25	Medicine	Neoplasia	Blood products	5	0	4.07	0	ME	
	Hawkins AE	J Virol Methods	01/06/1996												
5	CDC	MMWR	12/04/1996	USA	23	4	Dialysis	CRF	Standard	14	0	70	0	SE	
6				USA	22	3	Dialysis	CRF	Multi-Vials	2	0	0.66	0	SE	
7				USA	22	2	Dialysis	CRF	Multi-Vials	7	0	5.34	0	SE	
8				USA	20	2	Dialysis	CRF	Multi-Vials	11	0	14.29	0	SE	
9				USA	19	4	Dialysis	CRF	Multi-Vials	4	0	9.76	0	SE	
10	CDC	MMWR	14/03/1997	USA	10	5	Medicine	Diabetes	CBS	3	0	6.25	0	SE	
11				USA	12	2	Nursing	Diabetes	CBS	9	0	12.5	0	SE	
12	Parry CM	J Hosp Infect.	30/04/1997	UK	85	2	Dialysis	CRF	Blood products	1	0	-	0	SE	
13	Osterhaus AD	J Heart Lung Transplant	01/02/1998	Netherlands	24	145	Surgery	Transplant	TEB	20	0	7.81	0	SE	
14	Quale JM	Am J Med	01/10/1998	USA	24	11	Medicine	Diabetes	CBS	14	0	-	0	SE	
15	Kidd-Ljunggren K	J Hosp Infect.	01/06/1999	Sweden	28	1	Out-patients	Other	Multi-Vials	2	0	4.26	0	ME	
16	Hutin YJ	Infect Control Hosp Epidemiol.	20/11/1999	USA	43	4	Dialysis	CRF	Multi-Vials	6	0	33.33	0	SE	
17	Webster JM	Lancet	01/07/2000	UK	29	13	Out-patients	Other	Multi-Vials	29	0	9.23	0	ME	
	No Authors	Commun Dis Rep CDR Wkly.	27/02/1998												
18	Petrosillo N	J Clin Microbiol.	01/08/2000	Italy	29	6	Medicine	Neoplasia	Multi-Vials	11	9	9.32	81.82	ME	
19	Khan AJ	Infect Control Hosp Epidemiol.	01/06/2002	USA	30	5	Nursing	Diabetes	Undefined	6	1	12.73	16.16	ME	
20	Comstock RD	Infect Control Hosp Epidemiol.	25/07/2004	USA	23	40	Out-patients	Other	Standard	31	0	3.95	0	SE	
	CDC	MMWR	26/09/2003												
21	Missale G	Eur J Immunol.	01/11/2004	Italy	-	2	Medicine	Other	Multi-Vials	5	0	9.26	0	ME	
22	De Schrijver K.	Acta Clin Belg	01/03/2005	Belgium	22	4	Nursing	Diabetes	CBS	5	2	5.32	40	SE	
23	CDC	MMWR	11/03/2005	USA	22	16	Nursing	Diabetes	CBS	11	0	6.25	0	SE	
24				USA	13	9	Nursing	Diabetes	CBS	8	0	61.54	0	SE	
25				USA	15	6	Nursing	Diabetes	CBS	15	2	10.49	13.33	SE	
26	Samandari T	Infect Control Hosp Epidemiol.	01/09/2005	USA	43	24	Out-patients	Other	Standard	38	0	3.65	0	ME	
	CDC	MMWR	26/09/2003												
27	Dreesman JM	Epidemiol Infect.	26/01/2006	Germany	48	9	Nursing	Other	CBS	19	0	12.26	0	ME	
28	Fisker N	BMJ	11/02/2006	Denmark	-	14	Medicine	Neoplasia	Multi-Vials	7	0	3.45	0	ME	
29	Bracho MA	J Clin Microbiol.	01/04/2006	Spain	48	3	Surgery	Neoplasia	Undefined	2	2	-	100	ME	
30	Kondili LA	Eur J Clin Microbiol Infect Dis.	25/08/2006	Italy	41	17	Dialysis	CRF	Undefined	3	0	6.25	0	ME	
31	Rosenheim M	Gastroenterol Clin Biol.	30/11/2006	France	150	124	Surgery	Transplant	TEB	86	0	11.17	0	ME	
32	Redd JT	J Infect Dis	01/05/2007	USA	62	4	Surgery	Other	Undefined	1	0	12.50	0	ME	
33	Ramalingam S	J Clin Virol	07/09/2007	UK	138	2	Dialysis	CRF	Undefined	2	0	-	0	ME	
Synopsis of analyzed outbreak including bibliographic references and the most relevant epidemiologic parameters (SE=standard epidemiology; ME=molecular epidemiology. CFR= chronic renal failure; CBS= capillary blood sampling; TEB= transvenous endomyocardial biopsy; UK= United Kingdom; USA= United States of America; CDC= Centres of Diseases Control and Prevention; AR= Attack rate; FR= fatality rate; HCS=Healtcare setting Pub.Date= date of publication, time to pub= time in months since the end of the outbreak and the publication). 
